# An Irregular Graph Based Network Code for Low-Latency Content Distribution

**DOI:** 10.3390/s20154334

**Published:** 2020-08-04

**Authors:** Weiwei Yang, Ye Li

**Affiliations:** 1 National & Local Joint Engineering Research Center of Technical Fiber Composites for Safety and Health, School of Textile and Clothing, Nantong University, Nantong 226019, China; yangweiwei@ntu.edu.cn; 2 School of Information Science and Technology, Nantong University, Nantong 226019, China

**Keywords:** content distribution, low-latency, network coding, belief propagation

## Abstract

To fulfill the increasing demand on low-latency content distribution, this paper considers content distribution using generation-based network coding with the belief propagation decoder. We propose a framework to design generation-based network codes via characterizing them as building an irregular graph, and design the code by evaluating the graph. The and-or tree evaluation technique is extended to analyze the decoding performance. By allowing for non-constant generation sizes, we formulate optimization problems based on the analysis to design degree distributions from which generation sizes are drawn. Extensive simulation results show that the design may achieve both low decoding cost and transmission overhead as compared to existing schemes using constant generation sizes, and satisfactory decoding speed can be achieved. The scheme would be of interest to scenarios where (1) the network topology is not known, dynamically changing, and/or has cycles due to cooperation between end users, and (2) computational/memory costs of nodes are of concern but network transmission rate is spare.

## 1. Introduction

### 1.1. Background and Motivation

Low-latency content distribution to multiple users over a lossy and dynamic network is an important requirement in many emerging wireless applications. For example, in disaster recovery efforts, it is commonly required to disseminate content to a number of wearable devices or protective equipment in a timely and robust manner [1,2,3]. In these scenarios, random linear network coding (RLNC) [4] has potential as its coding nature enables fountain-like packet transmissions. Over a lossy network, RLNC can achieve reliable transmission without the need of packet acknowledgment. For example, RLNC can work atop user datagram protocol (UDP) similar to the quick UDP Internet connection (QUIC) protocol [5], which would considerably reduce the feedback cost and latency. Compared to conventional fountain codes such as the Raptor code [6], RLNC can further increase the throughput by allowing intermediate nodes of the network to recode packets. These benefits make RLNC quite attractive for fast content distribution.

One drawback of RLNC is its decoding computational/memory cost. When the number of source packets involved in coding, $N_{s}$, is large, the cost of using Gaussian elimination (GE) for decoding can be prohibitive, especially for wireless nodes. For $N_{s}$ in the order of tens or several hundreds, straightforward sparse RLNC such as [7,8,9,10] where many encoding coefficients are zero can be used. For larger $N_{s}$ of more than tens of thousands, which are commonly seen in content distribution, however, the decoding of the above schemes may again suffer performance deterioration because the number of nonzero encoding coefficients is still large. By splitting the packets into small generations of sizes much smaller than $N_{s}$, generation-based network coding (GNC) [11] can partly resolve this issue by only performing RLNC in the generation, and the multiple generations can be scheduled randomly throughout the distribution process (to avoid generation-by-generation notification). The *coupon collector’s problem* due to randomly scheduling the disjoint generations, which would cause many non-innovative (i.e., not linearly independent) coded packets being received by the users, can be alleviated by using overlapping generations [12,13]. Various overlapping GNC schemes have been proposed, including [14,15,16,17,18,19].

Two major decoding methods exist for GNC. One direction of research is to treat the encoding vector (EV) of each coded packet (from a generation) as a sparse vector over the $N_{s}$ original source packets (which is the same as in the straightforward sparse RLNC schemes), and then use sparse variants of GE to decode. This approach would succeed as soon as $N_{s}$ innovative packets (across all the generations) are received. However, the approach usually requires to pivot a sparse matrix of $N_{s}$ columns to exploit the sparseness of GNC, e.g., [8,20]. This, in programming implementation, still imposes high memory requirement for efficient random access of sparse matrix elements [21], otherwise the pivoting speed is significantly sacrificed. In practice, even for a moderate $N_{s}$ as a few hundreds, the decoding speed of sparse GE can be unsatisfactory [22].

The other general decoding method of GNC is *belief propagation* (BP) decoding, which was originally proposed in [12]. BP decoding only performs GE within each generation, and the decoded packets are subtracted from the remaining overlapping generations to help. The computational/memory requirement is significantly reduced as it is only in the magnitude of the generation size $\left(\ll N_{s}\right)$. The penalty is the *overhead* that the decoding may not succeed as soon as $N_{s}$ innovative packets are received because generations are not jointly decoded. However, this trade of overhead for computational/memory costs may be desirable in some scenarios, in particular where such costs are constrained but network transmission rate is spare, as commonly seen in the rapidly-growing Internet-of-Things (IoT) applications. This scenario is the main focus of the present paper.

With BP decoding, one major objective is to suppress the overhead. In this paper, we make the following contributions addressing this problem: (1) We propose a framework to design the GNC code via characterizing it as building an irregular bipartite graph, where the and-or tree evaluation technique [23] is extended to analyze its BP decoding performance, and (2) by allowing for non-constant generation sizes, we formulate optimization problems to design degree distributions from which generation sizes are drawn. Through extensive performance evaluations, we show that the code may achieve both low decoding costs and transmission overhead, as compared to using constant generation sizes [14,24].

### 1.2. Related Works

Using packet-level coding for content distribution has been widely studied in several previous works. One well-known work is the application of the Raptor codes for multimedia broadcast/multicast [25], which has been standardized in [26]. The Raptor code, however, is end-to-end. Since it does not support recoding at intermediate nodes, the throughput may not achieve the max-flow capacity over multi-hop links. In several recent works, e.g., [27,28,29], RLNC has been considered in content distribution in IoT scenarios. The works show that RLNC, possibly enhanced by recoding at intermediate nodes or via device-to-device communication links, can be effective for reducing content completion time. However, as mentioned, the supported number of packets is no more than several hundreds due to the high computational cost of RLNC.

It is noteworthy that in networks with known topologies, e.g., (parallel) line networks, there exists sparse RLNC schemes with low decoding costs and almost zero overhead, e.g., [17,30,31,32,33,34]. However, we note that these schemes do not apply to our interested scenarios where the network topology may be not known a priori, dynamically changing, and/or has cycles.

### 1.3. Organization

The remainder of the paper is organized as follows: Section 2 presents the system model and describes the encoding, recoding, and decoding operations. Section 3 models GNC schemes using irregular bipartite graphs. The and-or tree analysis technique is extended to study the BP decoding process on such graphs. In Section 4, a framework is presented that uses the analysis results for designing generation size distributions. The code design is evaluated in Section 5, and Section 6 concludes the findings.

## 2. System Model

We consider a network where a file consisting of $N_{s}$ packets are to be distributed from a source node *s* to a set of destination users via a lossy network. Each packet consists of *K* symbols from a finite field $F_{q}$ of size *q*. Links are modeled as Bernoulli erasure channels and the erasure probabilities are assumed to be fixed throughout the transmission. The system is discrete-time. At each transmission time, each node may send a packet to each of its downstream nodes. If no erasure occurs, the packet is received immediately by the neighboring node. Nodes are assumed to have no knowledge of the global network topology and do not exchange their buffer states information with other nodes. We assume that the destinations only acknowledge the source node upon the successful recovery of all $N_{s}$ source packets.

### 2.1. Precoding and Generation Constructions

Source packets are first *precoded* using a conventional fixed-rate erasure correction code. A total of $N=\left(1+\theta \right)N_{s}$ *intermediate packets*, denoted as $S=\{s_{i}\in F_{q}^{K},1\leq i\leq N\}$, are generated from the $N_{s}$ source packets supposing that a precode of rate 1/(1+θ),θ>0, is applied. The intermediate packets are then grouped into *generations*. For convenience, below we refer to packets in generations as intermediate packets even if the source packets are not precoded. Each generation is a subset of S. Assume that we construct *L* generations, $G_{l}=\left\{s_{1}^{\left(l\right)},s_{2}^{\left(l\right)},\ldots ,s_{|G_{l}|}^{\left(l\right)}\right\}$, 1≤l≤L, in which $s_{i}^{\left(l\right)}=s_{j}$ for some *j*. We assume that $\cup_{l=1}^{L}G_{l}=S$. We define $d_{R}≜\min_{l}\left|G_{l}\right|$, $D_{R}≜\max_{l}\left|G_{l}\right|$, and $a_{R}≜\left(1/L\right)\sum_{l=1}^{L}\left|G_{l}\right|$, where $a_{R}$ is the average generation size and is assumed to be an integer. The generations are said to be *equal-sized* if $|G_{i}\left|=\right|G_{j}|,\forall i,j$, or *unequal-sized* if $|G_{i}\left|\neq \right|G_{j}|$ for some i,j. The generations are said to be *disjoint* if $G_{i}\cap G_{j}=\emptyset ,\forall i\neq j$, or *overlapping* if there exists $G_{i}\cap G_{j}\neq \emptyset$ for some i≠j. For overlapping generations we have $\sum_{l=1}^{L}\left|G_{l}\right|>N$.

In a GNC code, we assume that the intermediate packets in each generation could be chosen at random from S as follows. With the generation sizes specified, the *N* intermediate packets are randomly permuted and then evenly partitioned into *L* disjoint subsets $D_{l}$ (we assume *L* to be a divisor of *N* throughout the paper; if that is not the case, we can append some null packets), one per generation, i.e., $D_{l}\subseteq G_{l}$. Therefore, $d_{R}=N/L=\left|D_{l}\right|,\forall l$. Such a partition ensures that each intermediate packet is present in at least one generation. After that, the remaining $|G_{l}\left|-\right|D_{l}|$ spots of $G_{l}$ is filled up by a random selection of packets from $S\backslash D_{l}$, where \ denotes set-minus.

### 2.2. Encoding and Recoding

The source node sends coded packets from generations on its outgoing links. For each transmission opportunity, one generation may be selected randomly or in a round-robin manner. The coded packet is then formed by combining packets belonging to the generation using RLNC over $F_{q}$. For $G_{l}$, a coded packet is in the form of $p^{\left(l\right)}=\sum_{j=1}^{|G_{l}|}g_{j}^{\left(l\right)}s_{j}^{\left(l\right)}$, where $g_{j}^{\left(l\right)}$ is the coding coefficient uniformly randomly chosen from $F_{q}$. $g^{\left(l\right)}=\left[g_{1}^{\left(l\right)},\ldots ,g_{|G_{l}|}^{\left(l\right)}\right]$ is referred to as the *encoding vector* (EV), and is delivered in the header of $p^{\left(l\right)}$.

At each node *j* other than the source node, *L* queues $Q_{j}^{l}$, 1≤l≤L are maintained to buffer received packets for each generation. A received packet is said to be *innovative* within $G_{l}$ if its EV is not in the span of the EVs of the existing packets in $Q_{j}^{l}$. We assume that received packets are processed such that non-innovative packets are discarded. In practice this may not be necessary, but the assumption simplifies the model.

Let $|Q_{j}^{l}\left(n\right)|$ be the number of *buffered packets* in queue *l* at time *n*. When a transmission opportunity is presented on an outgoing link (j,i) of node *j* to one of its neighboring nodes *i* at time *n*, a queue is chosen according to a scheduling strategy. We denote the index of the scheduled queue as $l_{ji}^{*}\left(n\right)$. A packet from $Q_{j}^{l_{ji}^{*}\left(n\right)}$ is then *recoded* using RLNC and sent to *i*. Since the recoding is linear, the recoded packet is still a linear combination of the intermediate packets of the selected generation, just with the EV updated. An array $\left[S_{ji}^{1}\left(n\right),S_{ji}^{2}\left(n\right),\ldots ,S_{ji}^{L}\left(n\right)\right]$ is maintained for each (j,i), where $S_{ji}^{l}\left(n\right)$ indicates the numbers that $Q_{j}^{l}$ has been scheduled for sending coded packets on (j,i) so far. We denote $P_{ji}^{l}\left(n\right)=\left|Q_{j}^{l}\left(n\right)\right|-S_{ji}^{l}\left(n\right)$ as the *local potential innovativeness* of the queue on the link. Here terms “local” and “potential” are used because the innovativeness is only from the sending-node’s perspective and does not incorporate knowledge of packet loss and reception events downstream from node *j*. We refer to arrays $P_{ji}\left(n\right)=\left[P_{ji}^{1}\left(n\right),P_{ji}^{2}\left(n\right),\ldots ,P_{ji}^{L}\left(n\right)\right]$, ∀(j,i) as the *buffer states* of node *j* at time *n*. If queue *l* is chosen, the value of $S_{ji}^{l}\left(n\right)$ is increased by one.

In this work, the following *maximum local potential innovativeness* (MaLPI) scheduling strategy [35] is adopted, which chooses the queue:

(1) $$l_{ji}^{*}\left(n\right)=\arg\max_{l}P_{ji}^{l}\left(n\right)$$

on (j,i) at time *n*. If more than one queue attains the maximum, one of them is randomly chosen.

**Figure 1:**
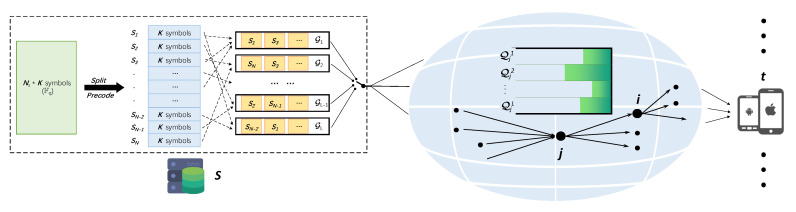
An overview of the system.

An overview of the system is summarized in Figure 1.

### 2.3. Belief Propagation GNC Decoding

The BP decoding is used at each destination node to recover the source packets from the received (re)coded packets, which are random linear combinations of the intermediate packets. The algorithm consists of two parts: The *inner decoding*, which recovers the intermediate packets and the *outer decoding*, which recovers the source packets from the intermediate packets. This paper focuses on the inner decoding.

The inner decoder decodes intermediate packets of each generation by solving a linear system of equations $A_{l}X_{l}=B_{l}$ using GE, where successive rows of $A_{l}$ and $B_{l}$ are the EVs and the coded *K* information symbols of the received packets that originate from $G_{l}$, respectively. In practice, on-the-fly GE [36] can be used for this task, which would progressively process packets and know immediately when $A_{l}$ becomes full-rank.

When one generation is decoded by on-the-fly GE, the decoded packets are subtracted from the received packets of other not-yet decoded generations that also contain the decoded packets. This process is referred to as *belief propagation*. If no decodable generations can be found after the subtraction, the node continues to collect packets until another decodable generation is found. When the number of decoded intermediate packets reaches a threshold, which depends on the precode rate, outer decoding begins and all the source packets are recovered using conventional erasure correction techniques.

Suppose that $N^{\prime }$ packets need to be received to completely recover $N_{s}$ source packets, we define the overhead $\varepsilon =\left(N^{\prime }-N_{s}\right)/N_{s}$. The GNC code should be designed to achieve low ε.

## 3. Irregular Graph Based GNC and BP Decoding Analysis

### 3.1. Graph Representation of GNC Code

Generation construction with *N* intermediate packets resulting in *L* generations is modeled as constructing a bipartite graph. The packets and generations correspond to two independent sets of vertices on the graph, referred to as *packet nodes* and *generation nodes*, respectively. An edge is created to connect a pair of packet and generation nodes if the packet is contained in the generation, so the total number of edges $E=\sum_{l=1}^{L}\left|G_{l}\right|$. A node is said to be of degree *i* if *i* edges are directly connected to the node. We say an edge is of packet-side degree *i* if its connected packet node is of degree *i* and of generation-side degree *i* if its connected generation node is of degree *i*, respectively. We denote, as a fraction of the *E* edges, the packet-side and generation-side degree *i* of the resultant bipartite graph as $\lambda_{i},1\leq i\leq L$ and $\rho_{i},d_{R}\leq i\leq D_{R}$, respectively.

Since generations are constructed at random, a GNC code can be viewed as a random graph drawn from an *ensemble* of graphs consisting of all bipartite graphs with the fractions of edges of packet-side and generation-side degree *i* being $\lambda_{i},1\leq i\leq L$, and $\rho_{i},d_{R}\leq i\leq D_{R}$, respectively. We refer to sequences $\lambda_{i}$ and $\rho_{i}$ as the *packet-side edge* and *generation-side edge* degree distribution, or by their generator polynomials $\lambda \left(x\right)=\sum_{i=1}^{L}\lambda_{i}x^{i-1}$ and $\rho \left(x\right)=\sum_{i=d_{R}}^{D_{R}}\rho_{i}x^{i-1}$, respectively. Equivalently, the graph can also be described by the *packet-diversity* distribution $\Psi \left(x\right)=\sum_{k=1}^{L}\Psi_{k}x^{k}$ and *generation-size* distribution $\Omega \left(x\right)=\sum_{d=d_{R}}^{D_{R}}\Omega_{d}x^{d}$, where $\Psi_{k}$ and $\Omega_{d}$ denote the probability that a packet node is of degree *k* and a generation node is of degree *d*, respectively; $\lambda \left(x\right)=\Psi^{\prime }\left(x\right)/\Psi^{\prime }\left(1\right)$ and $\rho \left(x\right)=\Omega^{\prime }\left(x\right)/\Omega^{\prime }\left(1\right)$ on the graph, where $\Psi^{\prime }\left(x\right)$ and $\Omega^{\prime }\left(x\right)$ are derivatives of Ψ(x) and Ω(x) with respect to *x*, respectively. We see that $\Omega^{\prime }\left(1\right)=1/\left(\sum_{i=d_{R}}^{D_{R}}\frac{\rho_{i}}{i}\right)$ is equal to the average generation size $a_{R}$.

### 3.2. Belief Propagation Decoding Analysis

The decoding of GNC codes includes two types of operations: The GE decoding of a generation and the subtraction of the decoded packets from other generations. Based on the graph representation, the BP decoding can be viewed as message passing between graph nodes. We use a modified and-or-tree technique of [23] to analyze the process, where the modification is due to the GE decoding of the generation nodes.

The graph is fixed throughout the transmission after generation construction. At the decoder side, each generation node is associated with a random number of received packets. We denote the probability that a generation node with μ received packets contains *k* innovative encoded packets as $p_{k,\mu }$, where k∈R={0,1,2,…,μ} and we refer to R as the *received ranks*. When RLNC is used, $p_{k,\mu }$ is equivalently the probability that a μ×k matrix (μ≥k) with elements uniformly randomly chosen from $F_{q}$ has rank *k*. The probability is [37]:

$$p_{k,\mu }=\left(1-\frac{1}{q^{\mu }}\right)\prod_{i=2}^{k}\left(1-\frac{q^{i-1}}{q^{\mu }}\right),\;1\leq k\leq \mu .$$

The term $\left(1-1/q^{\mu }\right)$ is the probability that the first column of matrix is not all-zero and $\prod_{i=2}^{k}\left(1-q^{i-1}/q^{\mu }\right)$ is probability that *i*-th column is not a linear combination of the previous i−1 columns. We have $p_{0,\mu }=1$ and $p_{k,\mu }=0$ for k>μ.

We define a binary message alphabet M={0,1}, where 0 and 1 stand for *unknown* (not decoded) and *known* (decoded) of a node on the graph, respectively. At the beginning of the decoding, every node on the graph sends *unknown* messages to its neighbors along the edges. Each generation node is associated with a received rank k∈R. The number of adjacent edges of a node carrying inputting unknown messages is referred to as the *unknown degree* of the node, denoted as $\varsigma_{p}$ and $\varsigma_{g}$ for packet nodes and generation nodes, respectively. Corresponding to the decoding process in Section 2.3, the message mapping rules on the graph is as follows: A generation node sends a *known* message on an adjacent edge if and only if its received rank *k* is larger than $\varsigma_{g}-1$, which means that the generation can be decoded by GE because there are *k* innovative packets while there are only $\varsigma_{g}\leq k$ unknown packets therein. A packet node sends known messages on its adjacent edges if and only if $\varsigma_{p}$ is smaller than its node degree, which means that at least one generation that contains the packet has been decoded.

The decoding is more easily explained and analyzed by the and-or tree evaluation technique [23]. By randomly choosing one edge of the bipartite graph that is uniformly sampled from the ensemble of graphs that are characterized by λ(x) and ρ(x), and expanding the graph starting from its connected generation node, we can obtain a subgraph being a tree with high probability [23]. We denote this subgraph as $P_{h}$, which is assumed to be obtained by expanding from a generation node to within distance 2h. Packet and generation nodes are at depths 0,2,…,2h−2 and 1,3,…,2h−1, respectively.

Let us consider the decoding of the root node of the $P_{h}$. Suppose that the subgraph was obtained by expanding from a generation node of degree *m* that has received μ packets. Let $u_{h}\left(m,\mu \right)$ denote the probability that it is not decodable. For $d_{R}\leq m\leq \mu$, we have $u_{h}\left(m,\mu \right)=1-p_{m,\mu }$ because generations can be decoded immediately if the number of their received innovative packets are larger than their degrees. We refer to this as *self-decodable*. For m≥μ+1, $u_{h}\left(m,\mu \right)$ is given in (2), where $z_{h}$ denotes the probability that an arbitrary packet node contained in the generation is sending an unknown message.

(2) $$\begin{array}{cc} u_{h}(m,\mu )= & \sum_{k=0}^{\mu -1}g\left(m,k,z_{h}\right)\left(1-p_{k+1,\mu }\right)\\ & +\sum_{k=\mu }^{m-1}g\left(m,k,z_{h}\right), \end{array}$$

where

(3) $$g\left(m,k,x\right)≐\left(\begin{array}{c} m-1\\ k \end{array}\right)x^{k}{\left(1-x\right)}^{m-1-k}.$$

The first term in (2) is the probability that the number of received packets of the generation node is larger than or equal to its unknown degree but the received rank is not equal to the unknown degree; the second term is the probability that the number of received packets is smaller than the unknown degree of the generation node.

Take all possible μ into account. Let $\eta_{m,\mu }$ denote the probability that the chosen root node is of degree *m* and associated with μ received packets. Note that $\eta_{m,\mu }$ is related to ρ(x) and the number of received packets for each generation. Let $y_{h}$ denote the probability that an arbitrarily chosen root node is not decodable by evaluating to within distance 2h on the bipartite graph, we have:

(4) $$\begin{array}{ccc} y_{h} & = & \sum_{m,\mu :m\leq \mu }\eta_{m,\mu }\left(1-p_{m,\mu }\right)\\ & & +\sum_{m,\mu :m\geq \mu +1}\eta_{m,\mu }\sum_{k=0}^{\mu -1}g\left(m,k,z_{h}\right)\left(1-p_{k+1,\mu }\right)\\ & & +\sum_{m,\mu :m\geq \mu +1}\eta_{m,\mu }\sum_{k=\mu }^{m-1}g\left(m,k,z_{h}\right)\\ & ≜ & f(z_{h},A), \end{array}$$

where the summations are over all possible (m,μ) pairs and *A* is a placeholder matrix consisting of probabilities $\eta_{m,\mu }$. The exact form of *A* will be specified in later sections when we design code.

Now we need to determine $z_{h}$. For h>0, since the subgraph $P_{h}$ is a tree, as explained in [23] we can evaluate $z_{h}$ based on subgraphs of $P_{h}$, $P_{h-1}$. The probability that a *d*-degree packet node beneath the root of $P_{h}$ sends unknown is as follows:

(5) $$v_{h}^{\left(d\right)}=\{\begin{array}{cc} 1 & d=1,\\ {\left(y_{h-1}\right)}^{d-1} & d=2,\ldots ,L, \end{array}$$

where $y_{h-1}$ is the probability that the root node in a subgraph $P_{h-1}$ is not decodable. The two cases in (5) correspond to (1) the packet node connecting to only one generation node (i.e., the root node of $P_{h}$), which is definitely not decoded, and (2) all other generation nodes connecting this packet node are not decodable, respectively. Therefore,

(6) $$z_{h}=\lambda_{1}+\sum_{d=2}^{L}\lambda_{d}{\left(y_{h-1}\right)}^{d-1}=\lambda \left(y_{h-1}\right).$$

Substituting (6) into (4), we have:

(7) $$y_{h}=f\left(\lambda \left(y_{h-1}\right),A\right).$$

This shows that, given fixed λ(x), ρ(x) and the number of received packets of each generation, the evolution of $y_{h}$, or in other words the decodability of each generation can be predicted. For h=0, the subgraph $P_{0}$ only contains the root generation node and its packet nodes. So $z_{0}=1$ and $y_{0}\leq 1$ corresponds to the probability that a randomly chosen generation is not self-decodable. The final value of $y_{h}$, denoted as $\delta ≜\lim_{h\to \infty }y_{h}$, corresponds to the smallest probability that the decoder can reach after going through all generations, or in other words, the fraction of generations that are not recoverable at the end of the BP decoding process.

For sources that are not precoded, all generations have to be recovered, so we need δ=0. This is infeasible because (7) is positive, which means that a not-precoded source is not guaranteed to be completely recovered given a fixed number of received packets. Interestingly, from another perspective this confirms that not-precoded GNC code would be affected by the “curse of coupon collector” [11].

For precoded GNC, choice of δ is straightforward because it is related to the precode rate 1/(1+θ). If there is a fraction δ intermediate packets that are not recovered by inner decoding, the packets ought to be recovered by outer decoding. This means that $N_{s}=\left(1/\left(1+\theta \right)\right)N$ source packets are to be recovered from any (1−δ)N intermediate packets. Therefore we have δ=θ/(1+θ). In the following we focus exclusively on precoded GNC codes.

For the sake of simplicity, we now omit the index *h* and denote the probability that a generation node is not decodable at any time as *y*, y∈[δ,1]. To ensure that the decoding process continues, we require:

(8) f(λ(y),A)<y, y∈[δ,1],

which means that the probability that a generation node is not decodable should be strictly decreasing until a fraction of (1−δ) generations are decoded. This inequality will be used in the rest of the paper.

### 3.3. Derivation of Ψ(x) and λ(x)

According to Section 3.1, we observe that Ψ(x) and λ(x) only depend on $a_{R}$ and $d_{R}$. The probability that a packet node connects to *k* generations using the generation construction of Section 2.1 is:

$$\Psi_{k}=(\begin{array}{c} L-1\\ k-1 \end{array}){\left(\frac{a_{R}-d_{R}}{N}\right)}^{k-1}{\left(1-\frac{a_{R}-d_{R}}{N}\right)}^{L-k}.$$

Therefore by some algebraic manipulations, we have:

(9) $$\Psi \left(x\right)=x{\left[1-\frac{\left(a_{R}/d_{R}-1\right)\left(1-x\right)}{L}\right]}^{L-1},$$

and using $\lambda \left(x\right)=\Psi^{\prime }\left(x\right)/\Psi^{\prime }\left(1\right)$, we have:

(10) $$\lambda \left(x\right)\approx (\frac{d_{R}}{a_{R}}+(1-\frac{d_{R}}{a_{R}})x)e^{-\left(a_{R}/d_{R}-1\right)\left(1-x\right)},$$

where the approximation is due to $\lim_{m\to \infty }{\left(1+\frac{1}{m}\right)}^{m}=e$.

### 3.4. Computational Complexity

The encoding complexity of the GNC code is $O(KD_{R})$ operations per encoded packet, where *K* is the number of symbols in the packet. For equal-size GNC codes, the decoder solves $L=N/d_{R}$ generations of equal-size $a_{R}$ by GE, so the decoding complexity is $O\left(L\left(a_{R}^{3}+a_{R}^{2}K\right)\right)=O\left(\gamma \left(a_{R}^{2}N+a_{R}NK\right)\right)$ to recover all generations, where $\gamma =a_{R}/d_{R}$, and is $O\left(\gamma \left(a_{R}^{2}+a_{R}K\right)\right)$ per decoded packet. The GNC code is therefore linear in *N* for fixed $d_{R}$, $a_{R}$, and *K*. For unequal-size GNC with average generation size $a_{R}$, some generations are larger than $a_{R}$. However, we show later that by carefully designing the generation-size distribution, the resultant GNC code may be decoded by only solving generations of an unknown degree of no more than $a_{R}$. Therefore, the decoding complexity of unequal-size GNC is upper bounded by equal-size GNC.

## 4. Irregular Graph Based GNC Design

### 4.1. Generation-Size Distribution Design

Based on the analysis of Section 3, we now design Ω(x) or ρ(x), from which generation sizes are drawn. From (7) and (4) we see that $\rho_{i},d_{R}\leq i\leq D_{R}$ are encapsulated in a joint distribution $\eta_{m,\mu }$. For convenience, we denote $\rho ≜[\rho_{d_{R}},\rho_{d_{R}+1},\ldots ,\rho_{D_{R}}]$. Unfortunately, $\eta_{m,\mu }$ is not easy to characterize because it also involves intermediary scheduling and erasures.

In this work, we resort to a heuristic simplification of $\eta_{m,\mu }$ to isolate ρ. That is, we only allow for non-zero $\eta_{m,\mu }$ at a specific μ to design ρ. We desire that such μ is smaller than $a_{R}$, so that the decoding cost can be reduced compared to if a fixed generation size of $a_{R}$ were used. The resulting problem corresponds to minimizing overhead for the case of when all generations receive the same number of packets. We note that this assumption may not be realistic given that the number of packets received per generation can hardly be equal due to random erasures. However, minimizing such μ can be seen as an approximation of minimizing the expected overhead. By applying the simplifications, we can rewrite (8) as:

(11) $$\begin{array}{c} \hat{f}\left(\lambda \left(y\right),\rho ,\mu \right)<y,\;y\in \left[\delta ,1\right], \end{array}$$

where,

(12) $$\begin{array}{c} \hat{f}\left(\lambda \left(y\right),\rho ,\mu \right)=\sum_{m=d_{R}}^{\mu }\rho_{m}\left(1-p_{m,\mu }\right)\\ \;\;+\sum_{m=\mu +1}^{D_{R}}\rho_{m}\sum_{k=0}^{\mu -1}g\left(m,k,\lambda \left(y\right)\right)\left(1-p_{k+1,\mu }\right)\\ \;\;+\sum_{m=\mu +1}^{D_{R}}\rho_{m}\sum_{k=\mu }^{m-1}g\left(m,k,\lambda \left(y\right)\right) \end{array}$$

and λ(y) is specified in (10).

Given fixed $a_{R}$, ρ can be optimized as the solution to the following problem:

(13) $$\begin{array}{cc} \underset{\rho }{\mathrm{minimize}} & \mu \\ \mathrm{subject}\;\mathrm{to} & \sum_{m=d_{R}}^{D_{R}}\rho_{m}=1,\\ & \sum_{m=d_{R}}^{D_{R}}\frac{\rho_{m}}{m}=\frac{1}{a_{R}},\\ & \hat{f}\left(\lambda \left(y\right),\rho ,\mu \right)<y,\;y\in \left[\delta ,1\right]. \end{array}$$

This problem can be solved by evenly discretizing the interval [δ,1] to generate multiple (e.g., M+1) inequalities in place of the single continuous one. For each point *y* at some multiples of (1−δ)/M, the inequality needs to be satisfied.

Denote the solution of μ as $\hat{\mu }$. Since $\mu \in \{d_{R},d_{R}+1,\ldots ,a_{R}\}$, we can obtain $\hat{\mu }$ by testing the problem feasibility with different μ, starting from the minimum possible value (i.e., $d_{R}$) up until the first feasible value of μ. It is observed that given λ(y) and μ, $\hat{f}\left(\lambda \left(y\right),\rho ,\mu \right)$ is a linear combination of $\rho_{d_{R}},\ldots ,\rho_{D_{R}}$ for each *y* in [δ,1], so (13) is a linear programming problem and can be solved using standard techniques.

### 4.2. Refinements to Generation-Size Distribution

For $\hat{\mu }$, the obtained ρ is supposed to be sufficient to ensure that the decoding is successful on average. However, some refinements still need to be made to ensure that the distribution works well in practice. The first refinement, similar to the design of ripple size in raptor codes [6], is to generalize constraints (11) by including a parameter $c_{\hat{\mu }}>0$, which represents the increment of decodabilities of other generations when a generation is decoded. Again, we can greedily search for the largest $c_{\hat{\mu }}$ from the initial value $c_{\hat{\mu }}=0$ such that (13) is feasible with known $\hat{\mu }$, i.e., enforce the probability increase as quickly as possible. Note that now the last inequality constraint is $\hat{f}\left(\lambda \left(y\right),\rho ,\mu \right)<y-c_{\hat{\mu }}$, and is still linear in ρ. Therefore, the optimal $c_{\hat{\mu }}$, which is denoted as ${\hat{c}}_{\hat{\mu }}$, is also the solution to a linear programming problem.

After obtaining ${\hat{c}}_{\hat{\mu }}$, an objective function can also be chosen to find a better ρ. A function that works well is the sum of $\hat{f}\left(\lambda \left(y\right),\rho ,\mu \right)$ on values of *y* discretized to generate the constraints. On one hand, from a performance point of view, minimizing $\sum_{y}\hat{f}\left(\lambda \left(y\right),\rho ,\mu \right)$ corresponds to maximizing the gap area between $\hat{f}$ and $y-{\hat{c}}_{\hat{\mu }}$, the latter is the upper-bound probability that a generation is not decodable at each stage of decoding. The larger the area is, the larger the portion of newly decodable generations we would have. On the other hand, the minimization is a least $l_{1}$-norm problem on ρ, which produces a ρ with a large number of zero components [38]. This is a good property because it would simplify generation construction in that only several generation sizes are possible even when the degree spread (i.e., $D_{R}-d_{R}$) is large. The generation-size distribution Ω(x) is then expressed in terms of ρ using the fact that $\Omega_{i}=a_{R}\rho_{i}/i,i=d_{R},\ldots ,D_{R}$.

## 5. Performance Evaluation

### 5.1. Outline of Design

We first outline the code design procedure. Suppose that we want to transmit *N* packets in *L* generations given $d_{R}$, $D_{R}$, and *q* and we require that the decoding recovers at least (1−δ) fraction of generations directly. Given the parameters, for different choices of $a_{R}$, we use the λ(x) specified in (10) and solve the refined (13) to obtain $\hat{\mu }$, ${\hat{c}}_{\hat{\mu }}$ and the corresponding Ω(x), from which we can sample generation sizes. For example, for $d_{R}=32$, $D_{R}=64$, $a_{R}=38$, δ=0.02, and $q=2^{8}$, we have $\hat{\mu }=33$ by solving (13), and ${\hat{c}}_{\hat{\mu }}=0.005$ for the first refinement. The Ω(x) after refinements is given by the following polynomial:

$$\begin{array}{cc} \Omega (x)= & 0.0058x^{33}+0.0991x^{34}+0.1495x^{35}\\ & +0.6341x^{39}+0.1109x^{40}+0.0007x^{64}. \end{array}$$

**Figure 2:**
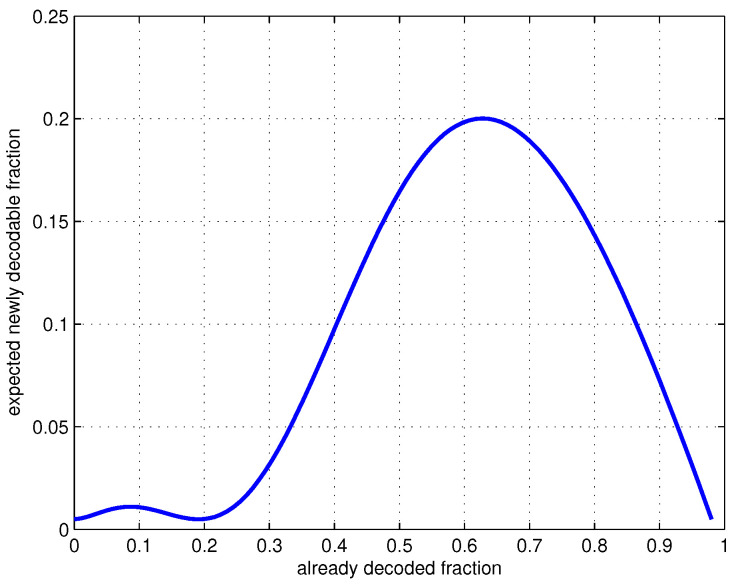
Expected newly decodable fraction of generations at various stages, $C=(32,64,38,0.02,2^{8})$.

In Figure 2, we plot the expected fraction of newly decodable generations ($x-\tilde{f}\left(\lambda \left(x\right),\rho ,\hat{\mu }\right)$) at various stages of the decoding process. This curve’s shape is typical for generation-size distributions considered here. The slowest period of the decoding process would occur at the beginning when few generations have been decoded. After that, the expected newly decodable fraction increases. This is an important feature in practice because it enables *avalanche finishing* when precoding is used. We will show this shortly. We note that values of *N* and *L* are not needed in the distribution design (as the analysis was on random ensembles), so Ω(x) is universal for the set of parameters $C=\{d_{R},D_{R},a_{R},\delta ,q\}$.

### 5.2. One-Hop Simulations

We now evaluate our code design in a single-hop setting by simulation and compare it with the disjoint chunking code (DCC) [11] and the *random annex code* (RAC) [14]. Our design is referred to as *irregular GNC* (iGNC) below. In single-hop networks, we do not need to consider buffer state because the source node has all its packets available. Packets are sent from each generation in a round-robin fashion to ensure that generations are scheduled evenly. Packets are erased with probability ϵ=0.2 over the link. The performance metrics of interest are the overhead and the associated computational cost. The latter is measured by bookkeeping the average number of finite field operations performed to decode each symbol of a source packet. The field size $q=2^{8}$ throughout the following simulations.

We first consider GNC without precoding to show that the designed iGNC can achieve a better overhead-complexity tradeoff. Assume that $N_{s}=$ 65,536 source packets to be transmitted, each contains K=1024 symbols from $F_{2^{8}}$, i.e., 64 megabytes (MiB) in total. We set the minimum generation size as $d_{R}=32$ and group packets into L=2048 generations. The simulation results are summarized in Table 1, where the bold values correspond to the minimum achieved overhead of the corresponding schemes. The average overhead and the number of operations per symbol needed in successfully decoding DCC, RAC, and iGNC with different $a_{R}$ are listed. The implemented decoder finishes decoding in less than 6 s on a Raspberry Pi 4B, achieving a decoding speed of about 10 MiB/s. (The implementation is not optimized. We note that this speed can be significantly improved by turning on single instruction multiple data (SIMD) of CPU (i.e., NEON for ARM) for finite field operations according to the measurement reports in [39]. However, we do not further explore this as the implementation optimization is not the focus of this paper.) On the contrary, this scale of $N_{s}$ would be prohibitive in terms of either decoding time or memory requirement for decoders other than BP, e.g., [20]. When $a_{R}=32$, RAC and iGNC reduce to DCC, in which no overlap is used. It is clear that DCC have the lowest computational cost but the largest overhead among all the configurations. For both RAC and iGNC, we see that there does exist a “sweet zone” when increasing $a_{R}$. The lowest achievable overhead and corresponding computation cost for each configuration is highlighted in boldface. It is clear from Table 1 that iGNC has much lower overhead and computational cost at the same time for all choices of $a_{R}$.

Results with precoding are also given in Table 1. When using a precode, we first encode $N_{s}$ source packets into $\left(1+\theta \right)N_{s}$ intermediate packets using a fixed-rate erasure-correction code. The generation construction process is then applied to intermediate packets. In our decoding process, there are (1−δ) fraction of generations recovered directly. On average, this leaves a total of $\delta La_{R}\left(d_{R}/a_{R}\right)$ intermediate packets that are not recovered, i.e., a δ fraction of intermediate packets. Here the multiplier $d_{R}/a_{R}$ is due to the overlap between generations. As a result, our precode should be chosen such that it recovers all source packets from intermediate packets with erasure rate δ, i.e., θ=δ/(1−δ)≈δ. We apply the same systematic LDPC precode as in the standard raptor codes ([40], Section 5.4.2.3). For $N_{s}=$ 65,536 and δ=0.02, S=1693 parity check packets are added such that the last 2% of packets can be recovered. It is noted that we need ⌈67229/32⌉−2048=53 more generations to ensure that each intermediate packet is contained in at least one generation.

It is seen that precoding is also helpful in DCC ($a_{R}=32$), and incurs almost no extra computational cost while reducing transmission overhead significantly. However, this improvement is not even competitive when compared to RAC and iGNC without precoding. By applying precoding to iGNC, we see that both overhead and computational cost can be further reduced. Specifically, for $a_{R}=38$, we can achieve overhead below 5%. The precoding is also beneficial to RAC, but its overhead and computation requirements are less favorable compared to that of iGNC for any choice of $a_{R}$.

Two points need to be highlighted here. First, we note that the benefit of precoding is only feasible when $a_{R}$ is smaller than the value at which the best overhead and computational cost is achieved in the non-precoding setting, i.e., 42 for RAC and 44 for iGNC in this example, respectively. It is because generation overlap can be viewed as a special type of zero-computation precoding in which we simply duplicate some packets. However, there exists an optimal amount of redundancy in combating coupon collector’s phenomenon. When the amount of redundancy from solely using overlapping has achieved its best overhead performance, adding more redundancy by applying precoding helps nothing but needs more generations to cover the check packets, which deteriorates the performance. Second, it is noted that the performance gap between RAC and iGNC with precodings is very small at the best $a_{R}$. The reason is essentially the same. Combining overlapping with LDPC precoding, a *cascaded precoding* design is actually obtained that is able to reduce much of the overhead caused by the coupon collector’s phenomenon. We emphasize that, as seen in Table 1, RAC is only comparable to iGNC when the best $a_{R}$ is known, which unfortunately is non-trivial to estimate. For any chosen value of $a_{R}$, however, iGNC tends to have lower overhead and computational cost all the time, which is a decisive advantage of it.

**Figure 3:**
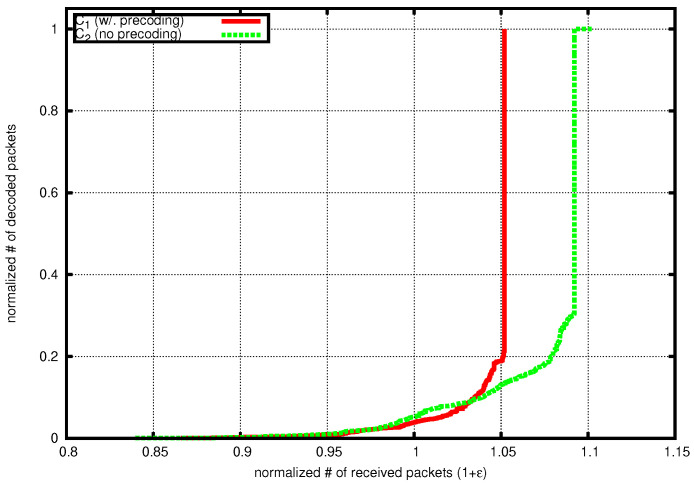
Example of decoding curve, $C_{1}=\left(32,64,38,0.02,2^{8}\right)$, $C_{2}=\left(32,64,44,0.02,2^{8}\right)$.

In Figure 3, we plot the decoding curves showing the number of collected packets versus the number of decoded packets for one decoding instance of precoded and not-precoded iGNC, respectively. Both numbers are normalized against the number of source packets. Parameters are chosen according to Table 1 such that iGNC achieves the lowest overhead. We see that the decoding curve of $C_{1}=\left(32,64,38,0.02,2^{8}\right)$ matches with the expected newly decodable fraction of generations during the decoding as shown in Figure 2. The code has spent most of its time collecting packets for recovering the first 20% of the source, and almost all packets are immediately recovered after that. In the case where no precoding is used, the decoding gets stuck when it is close to finishing and incurs a long tail in recovering the last few packets.

### 5.3. Network Simulations

We now evaluate the iGNC in two simple networks, namely the two-hop line network and the well-known butterfly network. Each hop of the two-hop link has equal erasure probability $p_{e}=0.2$, and each link of the butterfly network has equally $p_{e}=0.1$. The max-flow capacities of the two networks are known to be $C_{a}=0.8$ and $C_{b}=1.8$, respectively. Generations are scheduled in a round-robin fashion at the source node and MaLPI is used at intermediate nodes when recoding. $N_{s}=65,536$ source packets are transmitted. The same code parameters as in Section 5.2 are used. We examine the throughput rate, defined as the ratio of $N_{s}$ to the number of *network uses*, where each network use corresponds to that each link of the network transmits one packet. We compare the rates and computational costs of iGNC when RS and MaLPI are used, respectively. The results are shown in Table 2, where the highest achieved rates are marked as bold.

When $a_{R}=32$, the code reduces to DCC. It is clear from Table 2 that MaLPI achieves a higher rate. It is noted that the resulting throughput rate at best only achieves about 90% and 85% of the max-flow capacities of the two-hop and the butterfly network, respectively. The rate loss mostly comes from only making use of a *local* buffer state of each node in scheduling. As mentioned, a packet that is innovative from a sending-node’s point of view is not necessarily innovative for its downstream nodes, especially in networks where downstream nodes have multiple paths receiving packets. The proposed MaLPI scheme, however, is unaware of the issue because no coordination between nodes is available.

## 6. Conclusions

This paper has proposed using GNC codes with BP decoding for content distribution over lossy and dynamic networks. It was showed that GNC codes can be modeled as an irregular bipartite graph and its BP decoding performance can be analyzed through an extended and-or tree analysis. Using the analysis as the design tool, we managed to design degree distributions from which generation sizes are drawn through solving an optimization problem. Based on extensive performance evaluations, it was demonstrated that using non-constant generation sizes may achieve both a low decoding cost and transmission overhead compared to existing schemes where equal-size generations are used. We believe that the scheme has good potential in emerging wireless applications where end users of content distribution have limited computational/memory capacities.

For future works, it is of a great interest to evaluate the scheme in emulated/real-world network environment where links may have congestion and/or different propagation delays. Another interesting direction is to further suppress the overhead of BP decoding by incorporating more sophisticated operations such as inactivation decoding.

## Figures and Tables

**Table 1:** Comparison of codes at various $a_{R}$, $N_{s}=65536$, δ=0.02 and S=1693. RAC: Random Annex Code; iGNC: irregular Generation-based Network Code.

	RAC		RAC (Precoding)		iGNC		iGNC (Precoding)	
$a_{R}$	Overhead	Operations	Overhead	Operations	Overhead	Operations	Overhead	Operations
32	1.3226	32.41	1.1739	33.09	1.3226	32.41	1.1739	33.09
36	1.2566	36.42	1.0718	37.19	1.2349	36.41	1.0806	37.19
38	1.1977	39.45	**1.0522**	**40.36**	1.1888	39.19	**1.0497**	**40.13**
40	1.1578	43.28	1.1000	44.43	1.1439	42.31	1.0775	43.27
42	**1.1341**	**47.60**	1.1492	48.73	1.1339	45.05	1.0988	46.23
44	1.1722	52.07	1.2044	53.50	**1.1037**	**47.90**	1.1154	49.06
46	1.2255	56.83	1.2577	58.39	1.1043	50.51	1.1306	51.93
48	1.2804	62.03	1.3137	63.63	1.1147	53.15	1.1428	54.74
50	1.3332	67.26	1.3689	69.06	1.1253	55.98	1.1561	57.73

**Table 2:** Performance of iGNC in networks $N_{s}=65536$, δ=0.02. RS: Random Scheduling; MaLPI: Maximum Local Potential Innovativeness.

	Two-Hop Line Network ($C_{a}=0.8$)				Butterfly Network ($C_{b}=1.8$)			
	**RS**		**MaLPI**		**RS**		**MaLPI**	
$a_{R}$	**Rate**	**Operations**	**Rate**	**Operations**	**Rate**	**Operations**	**Rate**	**Operations**
32	0.5406	34.83	0.6307	33.81	1.0579	37.10	1.3452	34.35
36	0.5990	39.24	0.6908	38.09	1.1507	41.63	1.4724	38.69
38	0.6272	41.57	**0.7221**	**40.79**	1.2077	44.03	**1.5460**	**41.36**
40	**0.6525**	**44.74**	0.7055	44.11	**1.2382**	**47.35**	1.5295	44.73
42	0.6433	48.13	0.6892	47.50	1.2149	50.90	1.4897	48.31
